# Adipose Tissue and FoxO1: Bridging Physiology and Mechanisms

**DOI:** 10.3390/cells9040849

**Published:** 2020-03-31

**Authors:** Laura Ioannilli, Fabio Ciccarone, Maria Rosa Ciriolo

**Affiliations:** 1 Department of Biology, University of Rome “Tor Vergata”, Via della Ricerca Scientifica, 00133 Rome, Italy; laura.ioannilli@gmail.com; 2 IRCCS San Raffaele Pisana, Department of Human Sciences and Promotion of the Quality of Life, San Raffaele Roma Open University, Via di Val Cannuta 247, 00166 Rome, Italy; fabio.ciccarone@uniroma5.it; 3 IRCCS San Raffaele Pisana, Via della Pisana 235, 00163 Rome, Italy

**Keywords:** ROS, fasted state, adipogenesis, browning, mitochondria

## Abstract

Forkhead box O class proteins (FoxOs) are expressed nearly in all tissues and are involved in different functions such as energy metabolism, redox homeostasis, differentiation, and cell cycle arrest. The plasticity of FoxOs is demonstrated by post-translational modifications that determine diverse levels of transcriptional regulations also controlled by their subcellular localization. Among the different members of the FoxO family, we will focus on FoxO1 in adipose tissue, where it is abundantly expressed and is involved in differentiation and transdifferentiation processes. The capability of FoxO1 to respond differently in dependence of adipose tissue subtype underlines the specific involvement of the transcription factor in energy metabolism and the “browning” process of adipocytes. FoxO1 can localize to nuclear, cytoplasm, and mitochondrial compartments of adipocytes responding to different availability of nutrients and source of reactive oxygen species (ROS). Specifically, fasted state produced-ROS enhance the nuclear activity of FoxO1, triggering the transcription of lipid catabolism and antioxidant response genes. The enhancement of lipid catabolism, in combination with ROS buffering, allows systemic energetic homeostasis and metabolic adaptation of white/beige adipocytes. On the contrary, a fed state induces FoxO1 to accumulate in the cytoplasm, but also in the mitochondria where it affects mitochondrial DNA gene expression. The importance of ROS-mediated signaling in FoxO1 subcellular localization and retrograde communication will be discussed, highlighting key aspects of FoxO1 multifaceted regulation in adipocytes.

## 1. Introduction

The mammalian Forkhead box O (FoxO) family includes four members expressed nearly in all tissues: FoxO1 (forkhead in rhabdomyosarcoma, FKHR), FoxO3 (forkhead in rhabdomyosarcoma like protein 1, FKHRL1), FoxO4 (acute leukemia fusion gene located in chromosome X, AFX), and FoxO6 [1]. They are homologs of the *Caenorhabditis elegans* transcription factor Dauer Formation-16 (DAF-16) [1,2] and are characterized by the evolutionarily conserved forkhead domain, which is a winged-helix DNA binding motif that recognizes the core DNA motif TTGTTTAC [3]. Other conserved domains shared by the four FoxO members are the nuclear localization sequence (NLS), the nuclear export sequence (NES), and the *C*-terminal transactivation domain (TD). The transcriptional activity of FoxOs rules many cellular processes including differentiation, proliferation, metabolism, apoptosis, autophagy, and stress resistance in several tissues [4].

**Figure 1:**
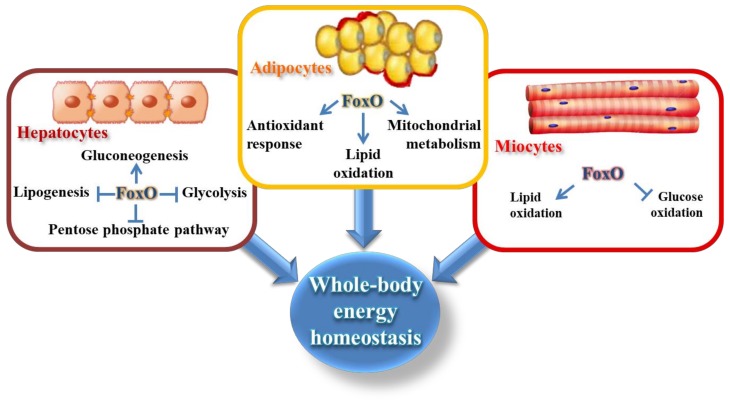
Scheme of the main metabolic pathways regulated by Forkhead box O (FoxO) proteins in the liver, adipose tissue, and muscle that are involved in the maintenance of systemic energy homeostasis.

FoxO1 is the first FoxO protein discovered in humans and it shows embryonic lethality in homozygous knockout mice [5]. FoxO1 plays an important role in many tissues being involved in the regulation of cell cycle and cellular metabolism [6]. In skeletal muscle, FoxO1 controls energy homeostasis, modulating glucose and fatty acid metabolism—e.g., by downregulating pyruvate dehydrogenase kinase 4 (PDK4) and upregulating the lipoprotein lipase (LPL), whereas in the liver it largely maintains whole-body glucose homeostasis—e.g., directly, by activating glucose-6-phosphatase (G6PC) and inhibiting glucokinase (GCK), or indirectly by suppression of miR-205-5p-mediated decrease of fed glucose levels [6,7,8,9]. However, FoxO1 is massively expressed in the adipocytes, where it organizes the transcription of genes related to adipocyte differentiation and transdifferentiation, oxidative stress defense, and lipid metabolism [10,11] (Figure 1). It regulates energy homeostasis through the modulation of the size of adipocytes and the adaptations to calorie intake [10,12]. Consistently, FoxO1 positively responds to increased mitochondrial reactive oxygen species (ROS) produced by starved adipocytes, determining the transcription of mitochondrial anti-stress response genes (mitohormesis). More importantly, FoxO1 is one of the most significant transcriptional factors involved in the insulin/insulin-like growth factor 1 (IGF-1) signaling pathway, responding to nutritional fluctuations via nucleocytoplasmic shuttling [13]. Notably, our recent study has also shown the existence of a mitochondrial FoxO1, under basal metabolic condition, that is delocalized by nutrient deprivation [13], which expands the dynamic role of this transcription factor in the control of adipocyte homeostasis. In this review, we are going to describe the multifaceted function of FoxO1 in adipocytes, highlighting its signaling role able to maintain energy and redox homeostasis during adipocyte differentiation and nutrient fluctuations.

## 2. Post-Transcriptional Regulation of Forkhead box O (FoxO) proteins FoxOs

**Figure 2:**
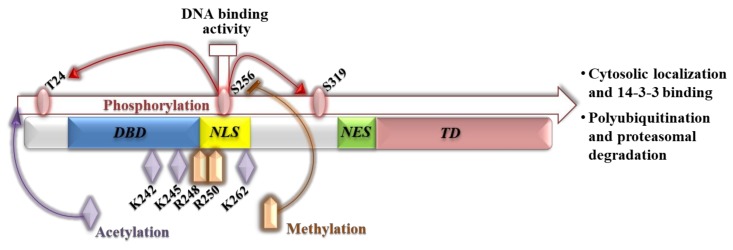
Scheme of human FoxO1 structure reporting the forkhead DNA-binding domain (DBD), the nuclear localization sequence (NLS), the nuclear export sequence (NES), and the *C*-terminal transactivation domain (TD). The main post-translational modifications of FoxO1 and their cross-talk are shown.

A wide range of external stimuli like IGF-1, insulin, cytokines, and nutrients control FoxOs activity through post-translational modifications such as phosphorylation, acetylation, methylation, and ubiquitination. These modifications alter protein subcellular localization, stability, DNA-binding properties and transcriptional activity [14]. In particular, the phosphorylation code is largely involved in FoxOs’ regulation and primarily determines the nucleocytoplasmic shuttling. Pioneering studies concerning metabolism, development, and longevity of the worm *C. elegans* have linked, for the first time, the insulin/IGF-1 signaling and the serine–threonine protein kinase B (AKT/PKB) with DAF-16/FoxOs transcriptional activity. The AKT/PKB signaling has been demonstrated to antagonize the DAF-16 activity during the inhibition of larval diapause (dauer arrest) [15]. Three conserved AKT phosphorylation sites are shared by all human FoxO proteins, except for FoxO6 that lacks the *C*-terminal site. As concerns FoxO1, the phosphorylation of Serine 256 (S256) by AKT decreases the binding affinity of FoxO1 to target DNA and triggers the phosphorylation of the other two sites, Threonine 24 (T24) and Serine 319 (S319) [16] (Figure 2).

Together with phosphorylation, acetylation also disables FoxO1 biological roles and these post-translational modifications show a fine-tuned cross-talk but non-overlapping functions [17,18]. The acetylation of three lysine residues (K242, K245, and K262) located at the *C*-terminal of the forkhead domain of FoxO1 by the cAMP response element-binding protein (CREB)-binding protein (CBP/P300) histone acetyltransferases weakens the DNA-binding affinity [4,19,20] (Figure 2). Consistently, the class of NAD-dependent deacetylase Sirtuins (SIRTs) positively regulates FoxO1 transcriptional activity [21,22,23]. SIRT1 directly interacts with a specific conserved motif (LXXLL) in the *C*-terminal domain of FoxO1 [24] and it has been shown to promote FoxO1 nuclear enrichment and transcription of glucogenic genes in hepatocytes overriding the phosphorylation-dependent nuclear exclusion of FoxO1 caused by growth factors [25]. SIRT1-mediated deacetylation of FoxO1 is also important for lipid hydrolysis and fat mobilization in differentiated adipocytes by activating the expression of the adipose triglyceride lipase (ATGL) during the fasting state [26].

Feeding/fasting state and oxidative stress are the principal events orchestrating the post-translational modulation of FoxO activity through phosphorylation, acetylation, and ubiquitination. These modifications largely affect the subcellular shuttling of FoxOs with consequences on the activation of downstream transcriptional programs [27].

### 2.1. Post-Translational Modifications of FoxO Affecting Nucleocytoplasmic Trafficking

The insulin signaling is evolutionarily conserved, thus it has been expectedly shown to impinge on FoxO activity also in humans. This pathway allows organisms to adapt to alterations in nutrient availability while maintaining systemic glucose homeostasis; for this reason, it is the key mechanism permitting FoxO protein to respond to feeding/fasting states. The binding of insulin/IGF-1 to their receptors elicits the activation of the intracellular kinase AKT which phosphorylates FoxO1 at T24, S256, and S319 [28]. Phosphorylated FoxO1 in this way undergoes inactivation and translocates to the cytoplasm where it binds to the adaptor protein 14–3–3 or is degraded by the proteasome [29,30]. Whereas AKT phosphorylation is typically a process that accounts for FoxOs cytoplasmic localization in response to insulin signaling, the phosphorylation of FoxOs by the mammalian Ste20-like kinase 1 (MST1) in response to oxidative stress has an opposite outcome. Specifically, FoxO3 has been shown to translocate to the nucleus to activate ROS-induced cell death in mammalian neurons after the phosphorylation of serine 207 (serine 212 in FoxO1) that disrupts the binding with 14–3–3 proteins [31]. In the same way, treatment with H_2_O_2_ or activation of the small GTPase Ral leads to c-Jun-N-terminal kinase (JNK)-mediated FoxO phosphorylation, which promotes nuclear translocation and activation of antioxidant cell response [32].

The interaction with the 14–3–3 proteins represents the key mechanism underpinning FoxO nuclear exclusion after AKT-mediated phosphorylation at the NLS because, by anchoring the phosphorylated FoxO protein already in the nucleus, they determine a conformational change that exposes the NES to the exportin 1, and thus, cytoplasmic localization [33]. Moreover, the binding of 14–3–3 induces steric interference with the forkhead domain causing a decreased DNA-binding capacity [34]. Once in the cytoplasm, the 14–3–3 proteins hide the NLS, preventing FoxO re-entry into the nucleus [28,33]. The importance of the phosphorylation state in the regulation of FoxO1 activity has been also confirmed by abrogating the dephosphorylation mediated by the protein phosphatase 2 (PP2A), which prevents nuclear localization and apoptotic response following cell death stimuli [35]. Moreover, the acetylation of FoxO1 by CBP/P300 has been shown to enhance the AKT-mediated phosphorylation causing nuclear exclusion [17], whereas overexpression of SIRT2 that deacetylates FoxO1 in the cytoplasm of pre-adipocytes promotes the nuclear translocation that accounts for the repression of adipocyte differentiation [36]. It has to be mentioned also that the methylation of FoxO1 can interfere with the phosphorylation of S256 by AKT. In fact, FoxO1 methylation occurs nearby the phosphorylation motif of AKT at conserved Arginine residues (R248 and R250) and is catalyzed by the Protein arginine methyltransferase-1 (PRMT1) [37], the silencing of which enhances FoxO1 cytoplasmic localization and proteasomal degradation (Figure 2) [37]. FoxOs, like many other proteins, are ubiquitinated to be degraded by the proteasome, but the monoubiquitination has the opposite effect and increases FoxO nuclear localization, inducing transcriptional activity [38]. SKP2, an oncogenic subunit of the SKP1/CUL1/F-box protein ubiquitin complex, promotes the degradation of FoxO1 and this effect requires AKT-specific phosphorylation of FoxO1 at S256. Moreover, the expression of SKP2 inhibits the transactivation of FoxO1 and abolishes the inhibitory effect of FoxO1 on cell proliferation and survival [39]. Therefore, all post-translational modifications give a multifarious aspect to FoxO1, able to regulate its activity and subcellular localization in response to different stress conditions.

### 2.2. Post-Translational Modifications of FoxO1 Inside Mitochondria

**Figure 3:**
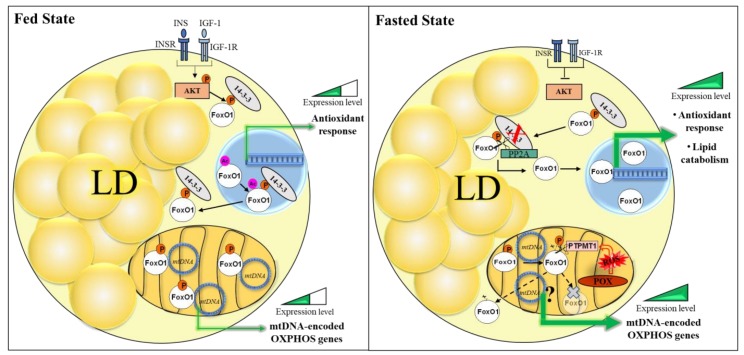
Subcellular distribution of FoxO1 driven by nutritive stimuli in white/beige adipocytes. Upon fed state, phosphorylated FoxO1 (p-FoxO1) localizes in the nucleus, cytoplasm, and mitochondria. The insulin signal activates the AKT pathway phosphorylating FoxO1. The phosphate group is an anchor for binding to 14–3–3 proteins in the cytoplasm. In the nucleus, p-FoxO1 is inactive, binds 14–3–3, and shuttles to the cytoplasm. In mitochondria, FoxO1 binds mtDNA mitigating the mtDNA-encoded gene expression. Upon fasted state, FoxO1 is dephosphorylated by PP2A in the cytoplasm, migrating to the nucleus to induce lipid catabolism (ATGL, LIPA) and antistress response genes (SOD2, UCP1). The fasted state induces proline oxidase/dehydrogenase (POX/PRODH) that produces mitochondrial ROS that activates PTPMT1. The dephosphorylation permits mitochondrial FoxO1 exclusion and increased mtDNA-encoded genes expression. Then, FoxO1 could be degraded or recruited to the cytoplasm/nucleus.

Interestingly, the phosphorylated isoform of FoxO1 at S256 has been also identified in mitochondria of adipocytes where it resides under physiological conditions. Contrarily to what happens in the nucleus, the abundance of nutrients preserves FoxO1 inside the mitochondria to fine-tune the transcription of mitochondrial DNA encoded genes [13]. On the other hand, fasting activates protein tyrosine phosphatase mitochondrial 1 (PTPMT1) to dephosphorylate FoxO1, determining a decrease of the FoxO1 mitochondrial pool (Figure 3) [13]. Nevertheless, no definitive conclusion on the destiny of mitochondrial FoxO1 upon nutrient deprivation has been drawn: it could be engaged to replenish the cytoplasmic/nuclear pool or selectively degraded by mitochondrial proteases [13]. It has been demonstrated that ROS produced by the fasted state specifically increase the PTPMT1 expression, but this phenomenon cannot be recapitulated by a general increase of oxidative stress, as the treatment with H_2_O_2_ [13]. Consistently, alternative ROS sources, such as H_2_O_2_ and rotenone, have different effects also on FoxO1 mitochondrial localization [13]. This indicates that the mitochondrial enrichment of FoxO1 is finely controlled and whether the outcome upon different prooxidant conditions depends on phosphorylation or other post-translational modifications of FoxO1 deserves further investigations.

## 3. FoxO1 Signaling in Adipose Tissue

Adipose tissue has the main function to store energy inside adipocytes in the form of neutral lipids during excess nutrient conditions and to release them by lipolysis during fasting, to guarantee whole-body energetic homeostasis. Indeed, adipose tissue is a very dynamic organ that rapidly readapts under many physiological stimuli, by changing number, size, metabolic and endocrine functions of adipocytes [40]. Several adipose tissue-resident cells, including immune cells and mesenchymal stem cells of the stromal vascular fractions, are necessary to support the remodeling of adipose tissue assuring clearance of dead adipocytes, adipogenic differentiation, and proper vascularization [41,42]. Adipocytes are dispersed in two main adipose depots: the white adipose tissue (WAT) and the brown adipose tissue (BAT). WAT is highly widespread in the human body and can be subclassified in the visceral WAT (vWAT), which is close to the internal organs, and the subcutaneous WAT (sWAT) [43]. BAT, instead, seems to have a more defined localization, extending in the cervical-supraclavicular area. BAT has been believed to be typical of infants until a decade ago, when nuclear medicine imaging demonstrated its persistence also in adult humans [44,45]. Each type of adipose tissue has a distinct physiological role. Although white adipocytes inside both WAT types have the main function of triglyceride storage in a single unilocular lipid droplet [46], sWAT also accounts for thermal insulation and dermal defense against infections. Moreover, vWAT expansion is commonly associated with metabolic disorders, such as cardiovascular disease and diabetes [46,47] differently from what observed for sWAT. This aspect is justified by differences in the metabolic and hormonal profiles; for example, lipolysis in sWAT is adequately inhibited by insulin, with respect to vWAT [48]. Moreover, vWAT is responsible for the release of adipokines and pro-inflammatory molecules that are associated with insulin resistance [48,49].

On the other hand, BAT is not implicated in energy storage, but it is specialized in the control of non-shivering thermogenesis. The brown adipocytes contain high numbers of mitochondria, that confer the typical brown color, and multilocular lipid droplets [46]. The mitochondria of brown adipocytes are characterized by high levels of the uncoupling protein 1 (UCP1), which dissipates the proton gradient to generate heat rather than ATP [46,50].

The third type of adipocytes, characterized by an intermediate phenotype between white and brown adipocytes, is mainly interspersed in subcutaneous WAT. These are known as “brite” or “beige” adipocytes which could develop a thermoregulatory behavior while maintaining the energy reserve in lipid droplets, like white adipocytes. The beige adipocytes are activated in response to environmental cues, such as chronic cold exposure: this process is defined as “browning” of white adipose tissue [51].

Responding to insulin and glucagon respectively during feeding and fasted state, the adipose tissue activates precise molecular pathways able to modify the expression and subcellular localization of metabolic enzymes and transcription factors [52]. Feeding stimulates the lipogenic pathway and storage of triglycerides in adipocytes, while fasting induces the activation of the lipolytic pathway and promotes the breakdown of triglycerides and the release of free fatty acids from adipose tissue [40,53,54]. In this scenario, FoxO1 inactivation by insulin signaling is necessary for activating the lipogenic program in adipocytes, whereas, in the fasted condition, it transcribes genes involved in lipid catabolism [10]. Before describing in detail the molecular mechanisms by which FoxO1 controls adipocyte differentiation and the metabolic adaptations to nutrients availability, it has to be noticed that FoxO1 has also a crucial role in other adipose tissue-resident cells linking immunity and metabolism inside the adipose tissue. For instance, under long-term high-fat diet (HFD), the nuclear localization of FoxO1 in adipose tissue macrophages is increased due to oxidative stress and impaired insulin signaling and allows the infiltration of macrophages inside adipose tissue that contribute to insulin resistance [55]. Moreover, HFD increases the nuclear localization of FoxO1 also in adipose tissue-resident CD4^+^ T cells, where it downregulates the expression of genes involved in the activation of Th2 cells and their homing inside the adipose tissue, dampening in this way energy expenditure by brown and beige adipocytes [56]. All these findings demonstrate that FoxO1 modulation by several signaling cascades represents the master regulatory system bridging different stimuli to the physiological response of adipose tissue.

### 3.1. FoxO1 Involvement in Adipogenesis

Adipogenesis is the process through which fibroblast-like preadipocytes deriving from mesenchymal precursors differentiate into mature insulin-responsive adipocytes [57]. The differentiation induces drastic changes in cell shape: the mesenchymal precursors become committed preadipocytes, then the growth is arrested, and after that, the cell cycle re-starts as a mitotic clonal expansion, to produce a population of terminally differentiated white adipocytes [58,59]. The persistent inhibition of FoxO1 before induction of differentiation by the selective antagonist AS1842856 [60] or by a silencing procedure [61] suppresses almost completely the adipogenesis. Nevertheless, the role of FoxO1 in adipogenesis is complex and tightly tuned, as it can act as a promoter or inhibitor according to the steps of differentiation. In support of this, FoxO1 expression is promptly induced after stimulation of adipogenesis [62], but its activation state (judged by monitoring the dephosphorylation state) is not maintained during the whole adipogenic differentiation process [60]. FoxO1 is mostly inactive at the beginning of clonal expansion when cells re-enter the cell cycle, but it becomes activated when post-mitotic growth arrest occurs [60]. This is in agreement with the fact that constitutively active FoxO1 inhibits adipogenesis by activating the transcription of the cell-cycle inhibitor p21 [62]. The second round of inactivation largely coincides with the upregulation of PPARγ, which is the master regulator of adipogenesis and fat storage [60]. This pattern is noteworthy as FoxO1 has been shown to inhibit the expression of PPARγ [60,63], and even more to trans-repress PPARγ pathway interfering with promoter DNA occupancy of the receptor via direct protein–protein interaction [64]. PPARγ regulates both terminal differentiation and metabolism in mature adipocytes, by binding as a heterodimer with RXRα-DNA at PPAR response elements. FoxO1 readily disrupts the PPARγ–RXRα–DNA complex by direct binding to PPARγ [65], thus inhibition of FoxO1 may assure the PPARγ-mediated transcriptional program during adipocyte differentiation.

Based on this, the antagonizing FoxO1 function may be useful for overcoming dysmetabolic conditions associated with the insulin pathway. For instance, the haploinsufficiency of FoxO1 in heterozygous insulin receptor knockout mice or high fat-fed mice has been shown to restore the size of white fat cells, ameliorating insulin sensitivity [62,66]. Transgenic mice overexpressing the dominant-negative FoxO1 (the *C*-terminal transactivation domain is truncated) in WAT displayed ameliorated insulin sensitivity, glucose tolerance, and increased energy expenditure [12]. This augments fat mass, restoring adipocyte differentiation, and upregulates GLUT4 and adiponectin expression [12].

It is interesting to note that GLUT4 membrane translocation and nuclear exclusion of FoxO1 are parallel and independent events downstream of insulin/IGF-1 signaling in the terminal differentiation of adipocytes when GLUT4 permits glucose-dependent lipogenesis, whereas the absence of FoxO1 allows PPARγ transcriptional activity. This is corroborated by the fact that the translocation of GLUT4 to the cell surface is blunted, while the nuclear exclusion of FoxO1 is maintained in adipocytes with compromised insulin signaling (hyperinsulinemia) [67]. More specifically, FoxO1 is fully responsive to physiologically relevant insulin concentration in the insulin-resistant adipocytes, while GLUT4 is not [67]. The permanent nuclear exclusion of FoxO1 during hyperinsulinemia leads to the progression of adipocytes differentiation, repressing the lipolytic pathway and enhancing triglyceride accumulation [68]. In the long-term, impaired lipid utilization in adipocytes might have deleterious outcomes; in fact, enlarged adipocyte size has been correlated with changes in adipocyte function and metabolic disease [69]. Therefore, the difference in the sensitivity of GLUT4 and FoxO1 to insulin would lead to an uncoupling of insulin regulation of glucose transport and lipid storage in adipocytes [67].

### 3.2. FoxO1 Involvement in the Browning Phenotype

In addition to the regulation of WAT differentiation and homeostasis, FoxO1 acts as a negative regulator of both de novo differentiation of brown adipocytes and in the white-to-brown transdifferentiation of mature adipocytes. One of the earliest evidence showing that FoxO1 affects thermogenic BAT is that the overexpression of the dominant-negative FoxO1 in BAT increases oxygen consumption, energy expenditure, and mitochondrial metabolism by upregulation of UCP1 and PGC-1α [12]. Brown adipogenesis is a complex process that might be divided into four phases: preconfluent proliferation, confluent growth arrest, hormonal induction with clonal expansion, and permanent growth arrest with terminal differentiation [70]. The third phase is critical because the mature phenotype begins through the insulin signaling that, besides the AKT pathway that phosphorylates and inhibits FoxO1, induces also the activation of the RAS-ERK1/2 MAPK pathway that phosphorylates and activates cAMP response element-binding protein (CREB) [71,72]. Among the other mechanisms, the combined regulation of CREB and FoxO1 downstream of insulin signaling is necessary to block the expression of necdin [73], a crucial molecule showing inhibitory effects on brown adipocyte differentiation [74]. In particular, CREB actively reduces necdin expression, whereas FoxO1 is a positive regulator of necdin activity and, for this reason, is inactivated for permitting the progression of brown adipogenesis [73].

The evidence that FoxO1 can dampen the thermogenic program in adipose tissue has been also confirmed by a study centered on the “browning” process. In fact, during white-to-brown conversion stimulated by cold exposure or forskolin treatment (a pharmacological agent that raises intracellular cAMP levels [75]), FoxO1 undergoes inactivation by the co-repressor ZFP238 that, in this way, allows the expression of UCP1 and mitochondrial biogenesis-associated genes [76]. Moreover, FoxO1 activity has been demonstrated to induce autophagy during adipocyte differentiation which has been linked to the downregulation of UCP1 and upregulation of UCP2/3 [77]. This result correlates well with the notion that inhibition of autophagy leads to browning of white adipose tissue, which is manifested by an increased expression of UCP1 [78,79]. In particular, FoxO1 directly binds to the promoter of the transcription factor EB (TFEB), a key regulator of autophagosome and lysosome biogenesis, promoting its transcription during adipocyte differentiation. The use of the FoxO1 antagonist AS1842856 significantly reduced TFEB expression, and thus the induction of autophagy, with consequences on the coordinated expression of UCP1/2/3 during adipocytes differentiation [65,77].

## 4. FoxO1 Response to ROS in Adipocytes

ROS-mediated regulation of FoxO1 activity has been described in several cellular systems and relies on multiple molecular mechanisms including changes in protein interactors, phosphorylation state, and subcellular distribution [80]. The interaction of β-catenin with FoxO is favored by oxidative stress and contributes to enhancing FoxO transcriptional activity at genes involved in resistance to oxidative damage in mammalian cells and *C. elegans* [80,81]. Reduced FoxO1 phosphorylation at S256 is associated with augmented ROS production in MCF-7 cancer cells overexpressing the mitochondrial aconitase, with consequences on autophagy induction [82]. JNK and nuclear translocation of FoxO1 has been shown to occur in endothelial cells and follicular granulosa cells upon oxidative stress [83,84].

Hormonal stimulation of adipocyte differentiation in human adipose tissue-derived stem cells also results in a transient increase of intracellular ROS deriving from the mitochondrial activity and NADPH oxidase (NOX) activation. Consistently, NOX4 overexpression has been shown to trigger adipocyte differentiation by itself or to enhance the process after hormonal induction. The transient increase of ROS during adipocyte differentiation is accompanied by the upregulation of FoxO1, the transcriptional activity of which is necessary for mounting antioxidant response via the upregulation of superoxide dismutase 2 (SOD2), catalase, and Glutathione peroxidase 1 (GPx1) to avoid detrimental oxidative stress. In this way, ROS production is tightly balanced during adipogenic differentiation, as the enhancement of the hormone-stimulated process due to NOX4 overexpression is accompanied by no further ROS accumulation [85].

ROS generated by the redox adaptor protein p66Shc, which is known to activate membrane-bound NOX, to transfer electrons from reduced cytochrome C to oxygen and to downregulate antioxidant enzyme synthesis [86], were demonstrated to concur to insulin-dependent activation of AKT and FoxO1 translocation to the cytoplasm [70]. Although this mechanism has been mainly deepened in brown adipocytes, it can be reasonably extended to white adipocytes, as p66Shc-dependent ROS were shown to promote adipogenesis and triglyceride accumulation in both cell types, after insulin treatment [87].

The most prominent metabolic role of FoxO1 in adipose tissue is played under ROS produced by the fasted state where it is recruited inside the nucleus promoting the transcriptional network associated with antioxidant response (SOD2, UCP1) and lipid metabolism. In particular, FoxO1 has a pivotal role in controlling the transcription of two important lipases: ATGL and lysosomal acid lipase (LIPA) [11,68]. A synergic relation exists between these two enzymes in promoting lipid catabolism for whole-body energy supply: ATGL promotes triglyceride breakdown releasing diacylglycerol and fatty acids, whereas LIPA acts in a selective type of autophagy called lipophagy [88]. As concerns the activation of FoxO1 in starved adipocytes, the mitochondrial ROS were identified as the triggering molecules. More specifically, activation of the proline oxidase/dehydrogenase (POX/PRODH), a mitochondrial inner-membrane enzyme that starts proline catabolism, contributes to generate ROS under nutrient shortage [89]. In support of the relevance of ROS signaling in FoxO1 nuclear translocation in adipocytes and lipolysis activation were the results with the antioxidant *N*-acetylcysteine, which abrogated both phenomena [27,90].

More recently, the evidence that FoxO1 also resides in mitochondria of adipocytes has expanded the dynamic role of this transcription factor in the control of adipose tissue adaptation to nutrient availability [13]. FoxO1 changes its intra-compartmental localization between the nucleus, cytoplasm, and mitochondrion in response to ROS associated with nutrient fluctuations and other prooxidant sources. At the mitochondrial level, FoxO1 is present also as phosphorylated isoform in the organelle matrix of white/beige adipocytes to mitigate the expression of oxidative phosphorylation subunits encoded by the mitochondrial DNA under fed conditions [13]. Conversely, in response to starvation, FoxO1 disappears from mitochondria in a ROS-dependent fashion, as a consequence of PTPMT1 activation and dephosphorylation at S256 (Figure 3). Therefore, FoxO1 response to nutrient stress downstream of ROS-mediated signaling allows retrograde communication via subcellular redistribution. In this way, adipocytes avert energetic collapse by improving oxidative capacity and buffering the consequent oxidative stress via the enhancement of antioxidant defense.

## 5. Conclusions

Adipose tissue is generally considered a fat sink depot linked to obesity development; instead, less consideration is given to the fundamental physiological role it rules. Moreover, adipose tissue function and distribution undergo dramatic changes in functional capacity with aging, contributing to chronic low-grade inflammation and several other age-associated hallmarks, including cellular senescence or dysregulated hormonal and nutrient-sensing pathways.

Interestingly, FoxO family members are considered pro-longevity factors and most of the biological functions that FoxO1 exerts in adipocyte differentiation and metabolism fit well with this view also in fat cells. In particular, FoxO1 plays a central role in the responsiveness to nutrients availability, thus ensuring energy and redox homeostasis. Its proper role has been well established at a nuclear level, where it activates metabolic genes to improve the metabolic lipid profile and antioxidant response. More recently, it has been shown to fine-tune cellular adaptations to nutrient deprivation also at mitochondrial levels acting on mitochondria-encoded genes. FoxO1 is recognized as an “anti-obesity” factor able to improve adipose metabolism and fat mobilization upon its fundamental stimulus that is the lack of nutrients. Along with this, FoxO1, by finely modulating the adipogenesis process in white adipocytes, may contrast adipose tissue accumulation and hypertrophy. On the other side, although little evidence still exists, the counteracting effect of FoxO1 on the browning process disagrees with the anti-aging attitude. In fact, in the last decade, numerous papers have highlighted pro-health and pro-longevity functions of adipose tissue browning as a reliable way that can attenuate obesity-induced inflammation and insulin resistance.

FoxO1-mediated adaptations that re-establish the original homeostasis are largely dependent on diverse source-derived ROS. The involvement of FoxO1 is tangible both during adipocyte differentiation and in differentiated adipocytes. In the first case, the transient increase of ROS during adipocyte differentiation has been accompanied by the upregulation of FoxO1, which induces the transactivation of anti-stress response genes to counteract oxidative stress. In differentiated adipocytes, FoxO1 responds to nutritive fluctuations, changing its intracellular compartments and triggering the retrograde communication. The diverse subcellular localization of FoxO1 in response to nutrient stress determines the activation of adipocytes’ defense against the energetic collapse enhancing the oxidative metabolism while fighting oxidative stress. We can assume that FoxO1 is a junction point between the harmfulness (high doses) and positive signaling (low doses) of ROS.

Finally, FoxO1 plays a pivotal role in ameliorating fat metabolism, maintaining redox homeostasis and thus counteracting pathologies such as obesity and type II diabetes. In fact, when molecular cascades that ensure systemic metabolic homeostasis are impaired, the metabolic disease can develop and eventually give rise to other correlated diseases spanning from hepatic steatosis to cancer [40,51]. The studies developed so far pose a starting point for future investigations to collocate FoxO1 in a therapeutic research plan focusing on metabolic syndrome and the aforementioned diseases.
